# Improving IoT Botnet Investigation Using an Adaptive Network Layer

**DOI:** 10.3390/s19030727

**Published:** 2019-02-11

**Authors:** João Marcelo Ceron, Klaus Steding-Jessen, Cristine Hoepers, Lisandro Zambenedetti Granville, Cíntia Borges Margi

**Affiliations:** 1 DACS, University of Twente, 7522 NB Enschede, The Netherlands; 2 CERT.br, Brazilian National Computer Emergency Response Team, Brazil, São Paulo 05801-000, Brazil; jessen@cert.br (K.S.-J.); cristine@cert.br (C.H.); 3 UFRGS, Federal University of Rio Grande do Sul, Porto Alegre 91501-970, Brazil; granville@inf.ufrgs.br; 4 USP, University of São Paulo, São Paulo 05508-010, Brazil; cintia@usp.br

**Keywords:** malware, IoT, botnet, malware analysis, SDN

## Abstract

IoT botnets have been used to launch Distributed Denial-of-Service (DDoS) attacks affecting the Internet infrastructure. To protect the Internet from such threats and improve security mechanisms, it is critical to understand the botnets’ intents and characterize their behavior. Current malware analysis solutions, when faced with IoT, present limitations in regard to the network access containment and network traffic manipulation. In this paper, we present an approach for handling the network traffic generated by the IoT malware in an analysis environment. The proposed solution can modify the traffic at the network layer based on the actions performed by the malware. In our study case, we investigated the Mirai and Bashlite botnet families, where it was possible to block attacks to other systems, identify attacks targets, and rewrite botnets commands sent by the botnet controller to the infected devices.

## 1. Introduction

The increasing number of Internet of Things (IoT) devices, combined with their deficient security capabilities, have led to the proliferation of malware targeting IoT devices. The majority of IoT malware simply takes advantage of weak built-in defenses and factory default credentials to compromise devices and to turn them into a *bot*, i.e., a remote controlled device connected to a common control infrastructure [1,2]. When an IoT device joins a botnet, that device is used for different purposes, including launching Distributed Denial-of-Service (DDoS) attacks. Large-scale botnets based on IoT devices can perform powerful attacks capable of affecting large-scale systems over the Internet [3,4,5].

Different malware families that target exclusively IoT devices have been released, e.g., Mirai, Bashlite, Tsunami, Hide and Seek, BrickerBot, Luabot, and Hajime. Since the source code from distinct malware families, such as Bashlite and Mirai, has been exposed and publicly available, there has been an increase in the number of variants associated with those families [6]. A feasible way to analyze that amount of variants in a scalable manner is to use dynamic analysis [2]. In dynamic analysis, suspicious files are executed in an instrumented environment (sandbox) to study the malware behavior and detect its signature. However, the characteristics of IoT malware pose some challenges to the investigation process, such as to handle network traffic generated by the malware when executed in an analysis environment [7].

Similar to Internet worms, IoT malware are very active: as soon as they infect an IoT system, they perform different attacks over the Internet to identify vulnerable systems and compromise them. A simple solution may consider blocking all connections aimed to the Internet. However, this strategy can potentially block connections to the botnet’s command and control (C&C). Consequently, the bot will neither join the botnet nor receive attacks instructions, making the system used to investigate the malware incapable of observing the attacker intent.

In this paper, we introduce a methodology for handling the network traffic generated by IoT malware in an analysis environment. More than mitigate the network traffic generated by the sample, we exploit the flexibility of the network layer. Our approach aims to: implement effective mechanisms to block attacks, fingerprint the botnet C&C, monitor the communication channel, and manipulate instructions targeting the infected device.To investigate the feasibility of our approach, we also take advantage of Software-Defined Networking (SDN) concepts. SDN architecture enables the network programming by controlling the network layer that surrounds the sandbox. This flexibility allows the analysis environment to automatically adapts itself by providing the best setup for the malware that is is being executed in the malware analysis environment. In our evaluation, we analyzed the most widely known IoT malware families—Mirai and Bashlite. Thus, we demonstrate how the packet manipulation, containment policy, and traffic redirection can be used to enhance the results from IoT botnet investigation.

The remainder of this paper is organized as follows. In Section 2, we review related work. In Section 3, we discuss IoT malware characteristics. In Section 4, we describe our analysis approach. In Section 5, we present our prototype implementation. Next, in Section 6, we discuss the malware behavioral signatures and, in Section 7, we introduce our experimental analysis. In Section 8 we present the limitations of our approach and, in Section 9, we conclude this paper, presenting final remarks and future directions.

## 2. Related Work

The rise of attacks that use IoT devices as a platform to deliver an unprecedented DDoS attacks has highlighted the necessity to investigate such attacks. The literature shows many researchers aiming to investigate IoT malware. The Mirai botnet, for example, has received much attention from researchers since the well-known attacks performed in 2016 [3].

Antonakakis et al. [8] presented an extensible study where the botnet Mirai is investigated, showing what classes of devices were affected, as well as the DDoS attacks types and targets. Mirai was also investigated by Constantinos et al. [6] who analyzed the botnet components and observed how the DoS commands are performed by the infected devices. In our own research efforts, we also observed analysis of other malware families, often performed to specific samples and using a manual approach [9].

Meidan et al. [10] proposed a methodology to investigate IoT botnet by using machine learning techniques. This research presents an anomaly detection method which extracts behavior snapshots of the network and uses artificial intelligence to detect anomalous network traffic emanating from compromised IoT devices. The authors evaluated the solution using the IoT based botnets, Mirai and BASHLITE. Prokofiev et al. [11] presented a study where machine learning was used to detect Mirai botnet in the early propagation stage. As a result, it is possible to detect infected machines and avoid them receiving botnet instructions. Similarly, Meidan et al., using the solution proposed by Prokofiev et al., aimed to identify compromised IoT devices using network anomaly detection.

The IoT malware analysis investigation is used to improve the detection of compromised devices and mitigate attacks from them. Traditional malware analysis solutions could also be used to investigate IoT malware. However, when used in the IoT context, the traditional malware analysis solution present limitations. In particular, the support of multiple CPU architectures and fine-grained network access containment rules are features that require improvements. The project entitled IoTPot [12] tackles this problem by deploying an infrastructure to analyze malware families targeting Telnet-enabled IoT devices. IoTPot introduced the first sandbox designed for IoT malware that analyzes samples of different CPU architecture. IoTPot uses a sandbox that emulates OpenWrt [13]—a popular Linux distribution for embedded devices—and implements a particular network access control. This access control uses packet-rate policies to limit the traffic generated by the malware. Additionally, the connections targeted to port 23/TCP are directed to a local server in order to collect brute force attacks and study the malware propagation characteristics.

A similar solution is presented by Detux [14]. Detux is a multiplatform Linux Sandbox developed to run malware samples focusing on the network traffic analysis. As a result, it provides reports containing all connection attempts and their respective responses. Detux is an open source tool and does not provide intrinsic mechanisms to manage outbound network traffic. Thus, analyzing malware using Detux without any external containment mechanisms can potentially affect third party systems.

The automated analysis process remains an interesting research topic where there is room from improvement. In particular, the investigation process regarding IoT botnet involves to run the malware and enable it to connect to the botnet C&C. Once this connection is established, it is possible to receive commands from the C&C and monitor its attacks targets. However, as described, the IoT malware are very network active and continuously perform propagation attacks while connected to the botnet controller. Consequently, the side effect of running this type of malware is to impact third-party systems thought the propagation scans.

Existing solutions and investigations presented by both academy and industry overlook the network layer in as a way to scrutinize the botnet attacker intents. The majority of solutions tend to use the network layer to apply some kind of traffic limitation (rate limiting) and they do not explore the features as packet manipulation and traffic redirection. In this work, we fill this gap by programming the network layer of the analysis environment using the SDN architecture features. As a result, it is possible to use distinct access policies in the analysis environment based on the malware behavior which can include the attributes from packets (header and payload), i.e., not only the destination port and protocol as other solutions do.

## 3. IoT Malware

IoT malware refers to the class of malware that targets IoT devices, mainly embedded devices built upon a variety of CPU architectures, including MIPS, ARM, and PowerPC. This class of malware relies on the nature of IoT devices that come with limited system resources and where security mechanisms usually are neglected.

Despite dealing with constrained devices, IoT malware can be sophisticated. More recently, few malware families have implemented anti-analysis techniques, code obfuscation, and system conditional behavior [2]. Mirai, for example, observes the system aspects before contacting the botnet C&C. If the requirements are not satisfied, the malware pretends to connect to a fake C&C, preventing the real one from being disclosed. Multiple variants monitor the system running processes looking for a set of process names associated with well-known malware in order to neutralize a potential rival and save the limited shared resources [15].

Most IoT malware is coded in C language and cross-compiled to multiples CPU architectures. The binary contains all the necessary libraries (static build) to decrease external dependencies and to increase its execution chances in different devices. These binaries usually implement features associated with DDoS attacks, backdoor installation, brute force attacks, remote command execution, ransomware and cryptocurrency miners. Despite the variety of the IoT malware, the botnets are keeping extreme popular among them [2]. Mirai and Bashlite are developed particularly to create large-scale botnets designed to launch different types of volumetric attacks [3].

In general, an IoT malware propagates itself by scanning for vulnerable devices in order to compromise them and to install itself. The scanning process includes identifying target services and attempting to log in using different credentials (brute force attacks). A common mistake is to assume that only IoT devices directly connect to the Internet are exposed to those attacks. Many IoT devices implement a technology called Universal Plug and Play (UPnP) that can automatically negotiate port forwarding to internal network devices, which means that devices behind Network Address Translation (NAT) can be reached by scans if the gateway device has UPnP enabled [16]. In fact, those malware functionalities are common in diverse malware families, including the most notorious Mirai and Bashlite. In the next section, we underline the functionalities of those families that were used in our case study.

### 3.1. Bashlite

Bashlite is a malware bot family that infects Linux-based devices to launch DDoS attacks. This malware has different variants and is also known as Gafgyt, Lizkebab, Qbot, Torlus, and  LizardStresser [6]. The malware propagates using brute force attacks targeting Telnet service (23/TCP). In this process, the malware generates random IP addresses and attempts to get access to them using a set of built-in dictionary credentials.

The malware connects to a command and control (C&C) infrastructure using a custom protocol based on Internet Relay Chat (IRC) where the messages are exchanged in clear text through a TCP session. We describe in more details the custom protocol used by Bashlite in the Section 4.2. With a few differences between the variants, this malware provides a set of instructions that include messages to launch distinct types of DoS, to start a scan, to perform brute force attacks using a set of hard-coded passwords, and a mechanism to run arbitrary shell commands on infected machines.

### 3.2. Mirai

The Mirai malware family presents characteristics very similar to Bashlite. However, it has more sophisticated features to control the infected device and to support (DDoS) attacks. In particular, Mirai implements different volumetric attacks based on protocols such as TCP, UDP, and Generic Routing Encapsulation (GRE). These capabilities enable the malware to perform impressive DDoS attacks affecting high profile targets [4].

The Mirai propagation approach consists of continuous scans over the Internet for vulnerable devices. The early versions concentrated the scan to ports 23/TCP and 2323/TCP, and afterwards other ports were incorporated [16]. This propagation process involves brute force attacks using built-in malware credentials. However, in a few variants, the propagation scan process is performed using device vulnerabilities exploitation. The C&C infrastructure used by Mirai has substantial differences from other malware families, as presented by Antonakakis M.  et al. [8]. Besides the C&C controller, which sends instructions to the infected device, Mirai has an entity called *reporter* that is responsible for keeping track of successfully compromised devices. In brute force attacks, for example, the bot sends the identified credentials directly to the *reporter* host.

Another capability of Mirai family is to sense the running device in order to decide the actions to be performed. The malware has features to detect debuggers, evaluate the device running processes, and inspect execution parameters. Furthermore, this investigated Mirai variant, should be instantiated in the system with a particular file name (*dvrHelper*) and respective parameters based on system architecture, such as ./drvHelper telnet.mips. Otherwise, the malware poses a different behavior and does not contact the botnet C&C.

## 4. Architecture

The traditional malware analysis setup consists in running a malware sample in a segregated network with some sort of limited access to the Internet. In this dedicated environment, all the observed network traffic comes from the actions performed by the malware sample being executed in the sandbox. Hence, we propose a network layer that takes over the network communication by handling the connections between the malware and the other components of the analysis infrastructure. Based on the observed traffic, the solution can trigger the proper action in the analysis environment, such as, rewrite packet’s payloads and define the access policy. To achieve that, we defined a modular event-driven architecture based on the framework MARS [17] detailed in the Section 4.1.

**Figure 1:**
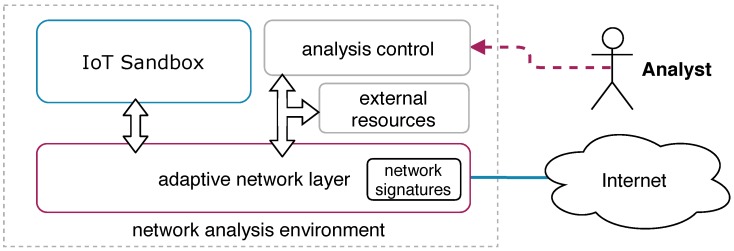
Adaptive network layer overview.

Figure 1 presents the building blocks of our approach and its relationship. The *IoT sandbox* is responsible for running the sample from the *malware repository* in the proper guest system and provides reports about the sample behavior during its execution. The module *analysis control* is responsible for controlling the execution flow, for submitting malware to the sandbox, and for providing the analysis report to the *Analyst*. We also defined a module called *external resources* that can be used to add additional services to the analysis environment. For example, we implemented a module that impersonates a botnet C&C used in our evaluation phase. That module acts as a botnet C&C implementing the initial handshaking from Mirai and Bashlite families. In some analysis scenarios, when the legitimate bot C&C is not accessible, it is possible to redirect the traffic to this module and collect communication details. Thus, in our approach, an analyst has the flexibility to add new modules to the environment in order to improve the analysis or to study a specific aspect of the malware’s family.

### 4.1. Adaptive Network Layer

As briefly described, our approach uses concepts from the SDN paradigm by using the framework MARS (MAlwaRe Analysis Architecture based on SDN) [18]. This solution uses SDN to manage the network flows implementing network traffic inspection in a centralized way. In that way, all traffic in the analysis environment can be handled from a single control point. MARS provides a set of APIs to interact with the network layer by performing the packet switching and by enabling packet inspection. We used this framework to design our adaptive network layer; however, also we implemented new building blocks on top of it to improve the IoT botnet investigation. The module *analysis control* (Figure 1), for instance, was specified to support the features proposed by our approach such as the ability to adapts the environment based on the malware behavior being analyzed.

This malware behavior is mapped in the system using a configuration file (*network signatures*) that characterize the network patterns (fingerprints) used on the adaptive network layer. Our system supports the use of many signatures, being customizable and extensible to different malware families. Hence, using a set of signatures (well-known traffic behavior), it is possible to design a custom setup configuration for a particular malware by improving the investigation. For example, a given malware has a specific propagation scan signature, then our approach can presume that this particular family is being analyzed and, therefore, can block this traffic destined to other devices.

Therefore, it is imperative to understand the malware families communication behavior in order to define the proper malware analysis configuration. Next, we discuss this network behavior from the evaluated malware families. Both families have multiples variants, but, for this study, we analyzed the respective publicly available source codes for Bashlite [19] and Mirai [15].

### 4.2. Network Behavioral Signatures

As previously described, the proposed adaptive network layer uses characteristics of the malware behavior to dynamically change the analysis setup configuration. In this section, we describe communication aspects from the analyzed families by discriminating the propagation scan and botnet C&C communication aspects.

#### 4.2.1. Propagation Scan

The propagation mechanisms implemented by the majority of IoT botnet are similar to the worm’s behaviors. Essentially, once they infect a device, they automatically perform scan and brute-force attacks in order to compromise other devices.

**A. Bashlite Scan:** The propagation process targets the service Telnet (23/TCP). The malware has a built-in dictionary with common usernames and passwords and uses them to connects to random IP addresses and attempts to log in. The analyzed sample [19], for instance, aims five usernames (root, admin, user, login and guest). Hence, as confirmed in our evaluation, the source code does not present any rate-limiting or a specific number of request. Our evaluation results show that this scan is a continuous process and is performed as soon the device is infected.

**Figure 2:**
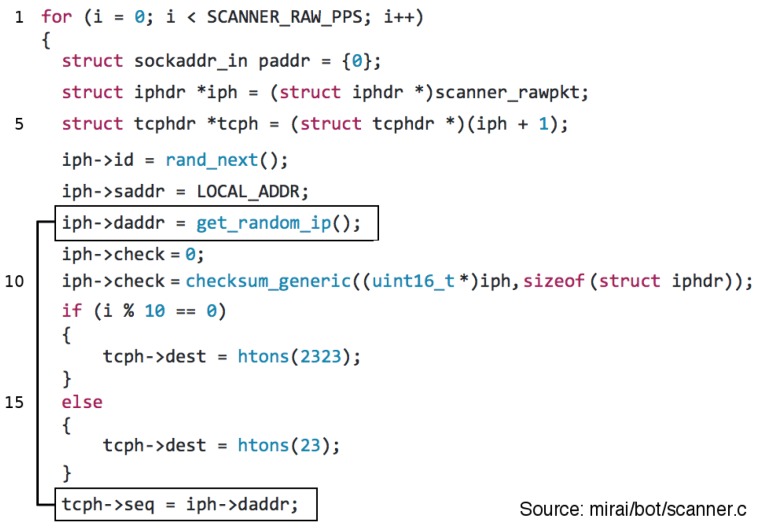
Mirai Scan Signature: The TCP scan packets are instantiated using the same value for the fields destination IP address and TCP Sequence number.

**B. Mirai Scan:** The propagation mechanisms implemented by Mirai consists of scanning a set of predefined TCP ports. Figure 2 illustrates the source code fragment associated with the malware scan capability. Based on that, we can observe (Line 11) that 90% of scans target port 23/TCP and 10% 2323/TCP. Although the scan traffic uses random parameters to avoid its identification and fingerprinting, it is possible to identify a peculiarity in the Mirai source code—highlighted in Figure 2—that was used to design a propagation scan signature [20].

These scan packets are crafted assigning a random value to the destination IP address and them assigning this same value to the field TCP packet sequence number (Line 19). Due to this characteristic, it was possible to distinguish the propagation scan attacks from the C&C regular traffic for this particular malware family.

#### 4.2.2. Botnet C&C Communication

**Figure 3:**
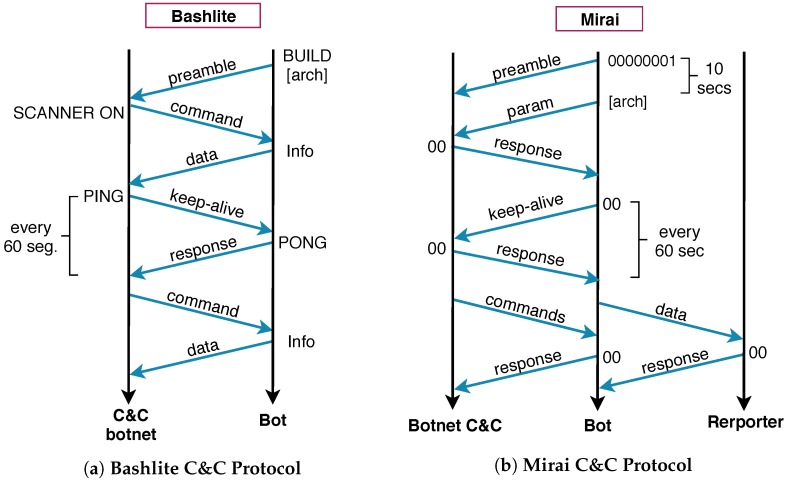
C&C Protocol: Message exchange process implemented by Bashlite and Mirai malware families.

IoT botnets must contact their respective C&C botnet controller in order to receive instruction and coordinate their behavior. Each botnet implements its own characteristics to enable this control, including protocol messages and communication scheme, as depicted in Figure 3.

**A. Bashlite C&C:** The communication protocol used by this malware family has a particular fingerprint. All messages are in clear text where the instructions from the C&C start with the character “!”. Thus, a feasible approach to detect the botnet C&C is to inspect the packet payload using that pattern. As soon as the C&C is identified, it is possible to restrain the communication to the botnet C&C and block attacks directed to third party systems.

The Bashlite communication protocol (Figure 3a) implements keep-alive messages exchanged every 60 s between the botnet C&C and the bot, e.g., PING and PONG messages. The other messages describe instructions for the infected device, including: SCANNER to perform 23/TCP scans; UDP to perform UDP flooding attacks; TCP to deploy TCP attacks; HOLD to hold a number of TCP connections for a specific period of time or delay attack for a specified duration; and GETLOCALIP to identify the local IP address.

**B. Mirai C&C:** The communication and control infrastructure implemented by the Mirai, as previously mentioned, is composed of three entities, namely the botnet C&C, the infected device (bot), and the reporter host. The communication scheme implemented by the Mirai (Figure 3b) uses a particular protocol based on hexadecimal messages. To connect to the botnet C&C, the bot has a peculiar handshake process where the first message is composed by the hexadecimal string 0x00,0x00,0x00,0x00,0x01. After 10 s, a literal that represents the system architecture is sent to the botnet C&C and, just after that, the C&C answers with an acknowledge message starting the heartbeat process. Hence, we used this specific handshaking string to identify the Mirai C&C.

The presented communication aspects were used to develop network signatures in our adaptive network layer. Consequently, it is possible to distinguish the malware families and trigger actions in the analysis environment. In fact, it is not necessary to study the malware source code to develop a signature; other techniques could be used to detect network pattern, as described by Van der Elzen [21]. Since our scope is to provide an environment to investigate malware samples, in this particular case, we opted to create the network signatures using the available source code, which makes the process easier.

## 5. System Implementation

**Figure 4:**
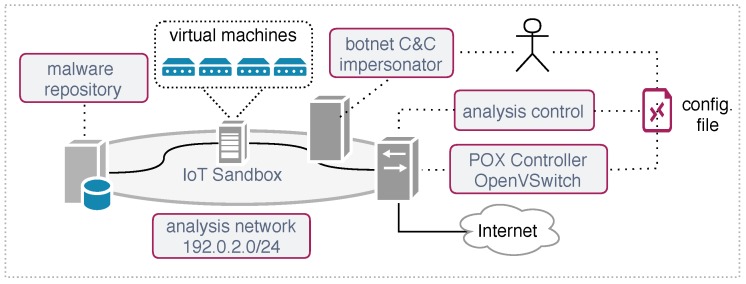
Malware analysis environment setup.

In this section, we describe our prototype implementation and the elements that comprise the malware analysis architecture depicted in Figure 4. We outline the setup configuration and the network behavior mapped using the previously described signatures.

### System Setup

The malware investigation was performed in a segregated network with Internet connectivity where all network traffic was controlled by our solution. Figure 4 depicts the analysis environment used in our evaluation.

The adaptive network layer was implemented using the SDN controller POX [22] and OpenVSwitch [23], which is a programmable software switch that can execute actions at the packet level. As discussed in Section 4.1, the controller is responsible for compiling the network configuration and for applying it to the OpenVSwitch. The network configuration (see Section 6 is defined by the Analyst and contains the topology, access policy and different actions that may be applied to the network packets.

The malware repository is responsible for maintaining the malware database and for providing the samples to the sandbox analysis. In particular, we used an heterogeneous malware database composed by samples collected at CERT.br Distributed Honeypots Project [24].

The element responsible for executing the malware was our extended version of Detux, as described in Section 2. The IoT sandbox evaluates the sample and instantiates the respective system platform using the QEMU [25] virtualization software. In this setup, we defined four virtual machines from distinct CPU architectures (x86, ARM, MIPS, and MIPSEL) running the Linux Kernel 2.6. Each sample runs in the sandbox for a period of 1440 min (24 h). We defined this execution time based on our experimental analyses, where this period was representative to collect C&C instructions commands and to determine the botnet characteristics.

The element *botnet C&C impersonator* emulates a botnet controller for Mirai and Bashlite IoT malware families. It was used to redirect specific connections performed by the malware running in the sandbox. More implementation details about this module are presented in Section 7.3. Next, we present implementation details describing how the malware behavior is mapped in our system.

## 6. Behavioral Signatures

Our system supports signatures that consider the full IP packet aspects, including UDP and TCP headers, and even the application-level protocols, such as HTTP messages. Those signatures are described in an XML based format and compiled in OpenFlow rules by our SDN controller. Listing  1 describes a fragment of the configuration file where the Mirai propagation scan signature is specified.

This configuration file comprises the general information regarding the analysis parameters and network signature expression. For example, the directive *sandbox-ip* describes the sandbox IP address. The *subnet* parameter defines the local network where the sandbox is installed; *bpf-expression* stands for Berkeley Packet Filter expression where the monitored packets are defined. The directives *proto* and *action* describe, respectively, the protocol used in the signature filter and the action that should be applied to the matched packets. *detux-args* describes parameters to the sandbox including the file name and respective arguments; *arch* defines the sandbox architecture that should be instantiated; and *time* represent the number of minutes that the sandbox will run the malware sample. In conjunction with the Mirai propagation scan signature, we used two other signatures: *Mirai C&C* and *Bashlite C&C*. Both signatures analyze the packets payloads looking for the patterns discussed in Section 4.2. Furthermore, those signatures can be combined to establish an execution control flow. Next, we detail how the signatures were combined to create a structured analysis process.

Listing 1: Mirai propagation signature: XML based configuration file used in the system setup configuration.

# Mirai propagation scan signature
sandbox-ip = 192.0.2.30
subnet     = 192.0.2.0/24
bpf-expression = "tcp[4:4] == ip[16:4]"
action = "drop"
proto = "tcp"
detux-args = "dvrHelper telnet.mips"
arch = "mips"
time = "1440 minutes"

### Execution Flow

We designed two execution flows based on Mirai and Bashlite network behavior. These flows were implemented in the SDN controller enabling the environment to automatically identify the malware family and customize its setup for proper operation. Next, we describe in more detail the two execution flows implemented in our evaluation.

**Figure 5:**
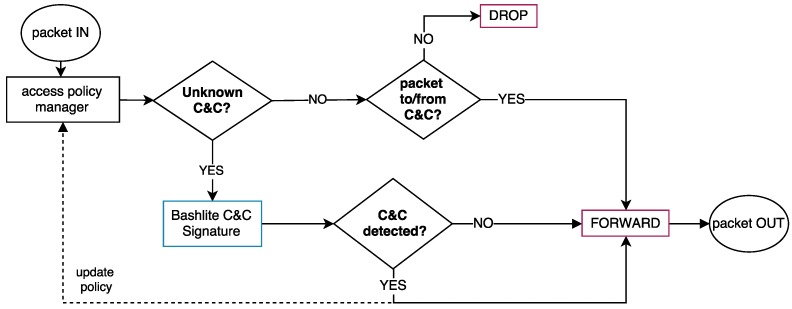
Bashlite’s execution flow: the bot initially establishes a communication with the C&C and then performs the propagation attacks scans.

**Bashlite**: This malware initially contacts the C&C and waits for instructions. As observed in our experiments, as soon as the majority of the variants connect to the C&C, they receive a scan propagation instruction, such as the SCANNER ON message. However, this propagation scan is only performed after the bot successfully contacts the C&C. Based on this behavior, we designed the execution flow—depicted in Figure 5—to handle Bashlite botnet communication.

When the botnet C&C signature is detected (*Bashlite C&C Signature*), the system dynamically updates the containment profile by allowing connections only to botnet C&C IP address and a set of local infrastructure services, such as DNS and NTP. Consequently, the attacks targeted to external systems—including the propagation scan—are discarded (*DROP*) by the SDN controller.

**Figure 6:**
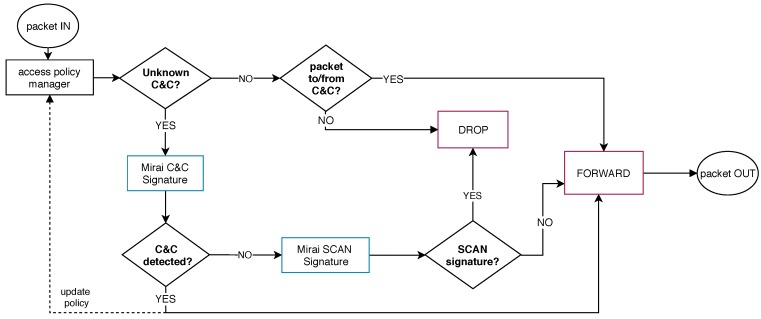
Mirai’s execution flow: The malware initiates the propagation scan process and simultaneously contacts its C&C.

**Mirai**: Implements a distinct initial behavior when compared to Bashlite. As soon as the malware runs, Mirai initiates the propagation process and, during this activity, the bot contacts its C&C using a particular handshaking mechanism, as described in Section 4.2. The Mirai analysis process should consider this behavior to avoid these propagation attacks from the botnet C&C communication. We designed this execution flow using two network signatures, as depicted in Figure 6.

When the SDN controller characterizes a propagation scan attack (*Mirai Scan Signature*), it promptly discards the packets and prevents them from being forwarded. Meanwhile, if the SDN controller identifies a network flows containing the *Mirai C&C Signature*, the system modifies the access policy by permitting flow to that C&C and a set of services, similar to those implemented in Bashlite execution flow process.

## 7. Case Study

We ran Mirai and Bashlite malware samples to better investigate the enhancement provided by our solution to IoT malware analysis process. The goal was to highlight how the responsive behavior can improve the analysis in terms of security and information completeness. We designed three experiments: in the first one, we focused on the network traffic containment, while, in the last two, we explored network flow manipulation features.

### 7.1. Experiment I: Network Access Containment

**Figure 7:**
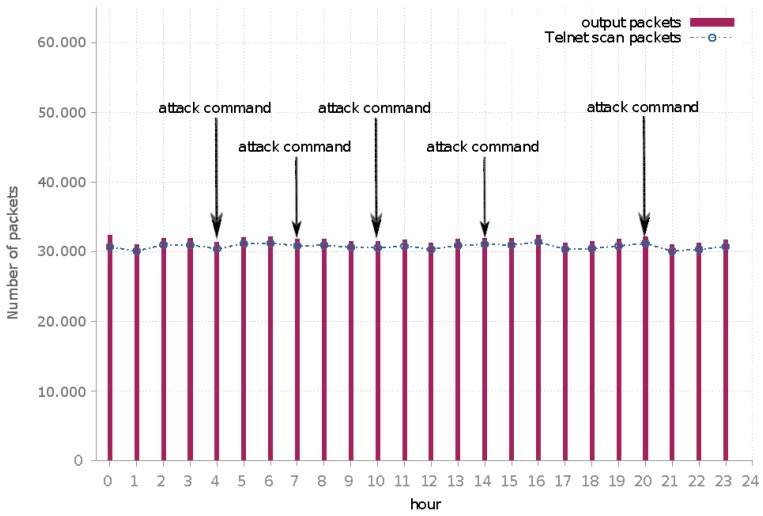
Bashlite egress traffic: the number of packets per hour generated by the analyzed malware.

This experiment investigated the ability of our system to distinguish the malware communication by blocking attacks and allowing the communication with the C&C. We observed the network behavior of Mirai and Bashlite samples running in our solution for a period of 24 h. Indeed, both malware families exposed a typical network behavior. Figure 7 summarizes the number of packets per hour generated by the analyzed Bashlite sample. The bars (red) represent the total number of packets in the analyzed sample in our analysis environment. The line (blue) represents how many of these packets are associated with propagation scan attacks (Telnet brute-force attacks).

As depicted, the propagation scan attack is very representative. After establishing the botnet C&C communication, this propagation scan traffic remained roughly constant during the entire analysis period (24 h).

The packets—not related to propagation scan—were associated with infrastructure services (DNS) and packets targeting the botnet C&C communication. This botnet communication was composed of keep-alive messages and attack commands.

In particular, during our analysis, we detected five attack commands issued from the botnet C&C to the infected bot. These commands were DDoS attack instructions targeting HTTP services (GET and POST) targeted to five distinct IP addresses. We could expect an increase in the number of packets as soon a DoS attack instruction was sent to the infected machine; however, this was not the case. This particular DoS attack (HTTP GET) required a fully established TCP session. In our system, the containment policy did not allow the infected machine to establish a TCP session with exception of the botnet C&C. The behavior shown through our system could successfully block attacks but enabled mapping their characteristics, such as destination targets and packet aspects.

**Figure 8:**
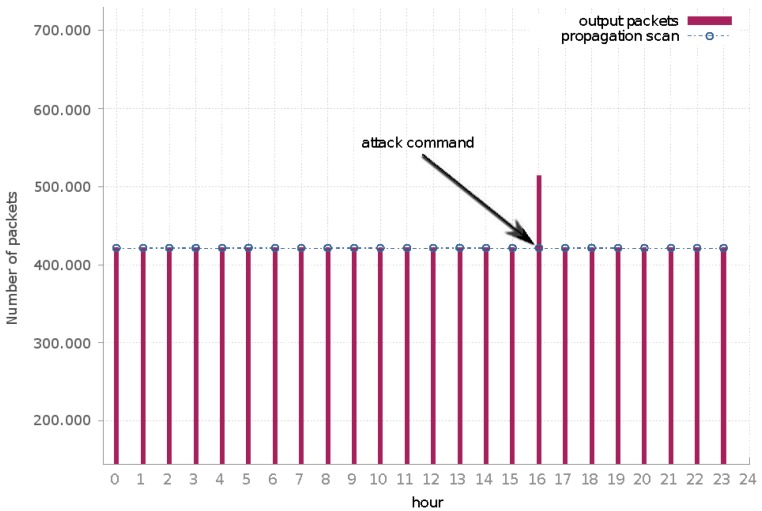
Mirai egress traffic: the number of packets per hour generated by the analyzed malware.

The Mirai analyzed sample had a similar network behavior, though the volume of egress traffic was substantially larger than Bashlite. Figure 8 underlines the network behavior in our system. Packets associated with the propagation scan process, similar to Bashlite, represented the majority of the network traffic. Here, these packets corresponded to attacks to ports 23/TCP and 2323/TCP aiming to perform attacks using malware embedded credentials. Due to the volume of such propagation scans, packets with the botnet C&C as the destination were indistinguishable in the graph baseline. In the communication targeting the botnet C&C, besides the keep-alive messages, we detected one attack instruction. This command instructed the infected bot to attack an IP address allocated to Russia using the *syn flood attack*. This attack was blocked by containment policy; however, as highlighted in *Hour 16* (Figure 8), we could observe the increase in the packet rate performed by the infected machine. The attack was performed for 10 s and issued a total of 92,419 packets.

In the performed analysis, the egress packet rate was constant at 120 packets per second. At the attack peak, the system handled 9361 packets per second, which reached our system maximum packet throughput rate.

The number of packets performed by Mirai scan was 13 times larger than Bashlite scan packets. Furthermore, we observed a similar behavior in both analyses: even during the attack instructions, the propagation scan packets were continuous and egress packet rate was quite constant.

Mirai and Bashlite are very network active and, without the proper containment, will affect other systems. Our approach effectively identified and blocked the attack commands from both malware families. The experiment was also successful in monitoring the communication with the botnet C&C providing valuable information regarding the attack’s targets.

### 7.2. Experiment II: Packet Manipulation

In this experiment, we studied the ability of our system to rewrite the different aspects of the malware’s generated packets. More than rewriting the packet’s header, we modified the packet’s payload to manipulate attack commands. To illustrate the process, we analyzed messages exchanged between a Bashlite bot and its respective botnet C&C.

The SDN controller could intercept messages and modify pre-defined botnet instructions. In particular, we implemented a signature to identify messages containing scanner instructions, such as the SCANNER ON message (see Figure 3a). When the SDN controller detected that instruction, the packet was intercepted, the payload was rewritten to SCANNER OFF, and then forwarded to the sandbox. This rewriting process inhibited the bot from performing the propagation scan attack as it was supposed to do with the SCANNER ON message. To illustrate this experiment, we present a fragment from a live experiment where that botnet instruction was rewritten in Listing 2.

Listing 2: Conversation between the infected device and Bashlite C&C where the instruction message SCANNER ON was rewritten to SCANNER OFF.

T 192.0.2.30:41828 -> 46.XX.XX.XX:666 [AP]
.[32mDEVICE CONNECTED .[31m-> .[37m192.0.2.30|.[32mTYPE .[31m-> .[37mSERVER.
 
T 46.XX.XX.XX:666 -> 192.0.2.30:41828 [AP]
  !* SCANNER ON.
 
T 46.XX.XX.XX:666 -> 192.0.2.30:41828 [AP]
  !* SCANNER OFF.
 
T 46.XX.XX.XX:666 -> 192.0.2.30:41828 [AP]
  PING.
 
T 192.0.2.30:41828 -> 46.XX.XX.XX:666 [AP]
  PONG.

This communication fragment shows the TCP packets payload exchanged between the sandbox (192.0.2.30) running the Bashlite malware sample contacting the botnet C&C (46.XX.XX.XX). Initially, we noticed a TCP conversation where the bot contacted its C&C using an identification banner. As soon this connection was successfully established, the botnet C&C sent an SCANNER ON message, which was intercepted by our SDN controller.

This particular message was rewritten and forwarded to the sandbox. Consequently, the bot did not execute the propagation scans but initialized the keep-alive messages exchanging process.

This packet payload rewrite capability, as shown, is an effective way to investigate botnet communication. By using the configuration file, an analyst can easily expand to other protocol messages. With the exception of encrypted communications, many other flows can be manipulated, including the rewrite of DNS messages and HTTP requests.

### 7.3. Experiment III: Bot redirection

In this evaluation, we investigated the ability of our solution to redirect network flows in a transparent way. In the previous experiment, we focused on the in packet payload manipulation; here, we tackled the TCP transport protocol.

This experiment aimed to redirect an infected bot to a botnet C&C under our control. As expected, malware samples embedded information regarding its botnet C&C. However, this botnet C&C may not be accessible or temporarily offline. Hence, to run this malware in an analysis environment will not reveal detailed information about the botnet join process, such as device identification and credentials used by bot to join the botnet C&C.

To tackle this problem, our solution enabled redirecting the requested botnet C&C to another one. With this capability, an analyst can forward the bot to an active botnet C&C. Furthermore, an analysis could emulate this C&C using the *botnet C&C impersonator* module (see Section 4). In this study, we deployed two tests. In the first one, we redirected a Bashlite sample to our impersonator module. To this end, we implemented this module using Perl, where it received messages from the bot and performed the keep-alive cycle.

By using this simple implementation, we analyzed a set of malware that does not have an active botnet C&C. In this manner, we were able to identify the bot initiation process characteristics. Thus, we collected distinct bot identification banners that expose the system host configuration aspects and could be used to identify the malware variant characteristics. Listing  3 illustrates the initial messages sent from the bots.

Listing 3: Initial messages sent from multiple Bashlite variants.

[Bash-Lite] [Connected [SERVER] [192.0.2.30]
SERVER Connected [192.0.2.30]
BUILD SERVER:[192.0.2.30]
[DEVICE CONNECTED] -> [192.0.2.30|TYPE -> SERVER]

An enhanced implementation of the *botnet C&C impersonator* module could implement other botnet messages, such as the attack instructions. For instance, it was possible to instruct the bot to attack a local service to identify the malware attacks characteristics. Furthermore, the *botnet C&C impersonator* could send unsupported messages to the bot to map its capabilities or even detect implementation faults.

In the second analysis, we redirected the malware to join in an illegitimate botnet, i.e., to contact to a different IP address from the one that was embedded in it. Our live experiment showed that this process is perfectly feasible because most IoT botnets are simple and do not incorporate authentication process. However, few botnet C&Cs, to avoid reinfection, restrict the number of connections based on source IP address. When multiples bots from the same IP address try to connect to the botnet C&C, it is common to receive disconnect messages such as DUP and KILLMYSELF. If necessary, an analyst could manipulate this behavior by rewriting those messages and avoiding the bot disconnection.

This experiment showed how packet redirection can be used to enhance the botnet communication. This feature has already been used in other works [26]. However, our solution does that in a transparent way, giving flexibility to the analyst to expand this configuration by composing it with other features previously described —packet payload rewrite, containment policy, and network signatures—in a centralized way.

## 8. Limitations

In dynamic malware analysis investigation, the malware is executed in the sandbox. Hence, the sandbox is a critical element in the analysis architecture. In situations where the malware does not properly run in the sandbox, whether for anti-sandbox techniques or lack of system requirements, this will affect the analysis results.

This is a limitation that impacts all systems based on sandbox tools and should be considered during the system implementation. Furthermore, being an SDN-based solution, our approach is also vulnerable to attacks targeting the SDN models. If the SDN controller is compromised, the malware analysis reports may be affected. In particular, the resources provided by our solution, such as packet manipulation and containment policy may also be fingerprinted by malware that senses environment characteristics. Even though this is not the scope of our research, it is fundamental to consider these aspects to map vulnerabilities in the system and improve its security.

## 9. Conclusions and Future Work

The security of IoT devices poses a significant threat to the Internet, since most of these devices do not implement the best security mechanisms to protect them from abuses. An increasing number of malware has explored the vulnerabilities of these devices to perform powerful attacks. Hence, to protect systems from such malware and improve the mechanisms of defense, it is essential to characterize the malware behaviors.

In this paper, we present an approach for analyzing IoT malware that can adapt itself to mitigate the network traffic generated by samples against other systems without affecting the quality of the results. Thus, using a centralized control provided by SDN, it is possible to manipulate the network flows generated by the sample and dynamically configure the analysis environment by modifying network topology, manipulate packet aspects, and enforce network containment policies. This integration between the malware analysis process and the network layer brought flexibility to investigate malware in distinct scenarios.

To evaluate our solution, we analyzed the Mirai and Bashlite malware families in three experiments. In the first experiment, which focused on network traffic containment, we showed that it is possible to distinguish the malware communication by blocking attacks and allowing the communication with the botnet C&C. In the second experiment, we exploited the capabilities of our system to rewrite packet payloads. As a result, we demonstrated how this capability can be used to modify the botnet instructions messages as well as the ability of our system to manipulate the network flows by forwarding a bot to an illegitimate botnet control infrastructure.

To succeed in those experiments, we investigated the IoT botnet behavior and used a network signature to characterize its behavior and trigger actions in the analysis environment. In fact, the use of network signatures provides flexibility to our system but, at the same time, it requires previous understanding of the malware characteristics. In our approach, an analyst can easily implement distinct setup configurations to determine malware characteristics and develop signatures. For instance, it is possible to analyze a sample in an environment using a restrictive access policy and, as soon as its network actions are revealed, customize the environment access policy.

As future work, we will investigate other IoT botnet behavior and provide the signatures associated with them in a public repository. In this way, these resources will be available to researchers to compose their own customized malware analysis process. Furthermore, the use of pre-defined configuration profiles could be extended and, consequently, improve the malware analysis process.
